# Gait Impairment and Upper Extremity Disturbance Are Associated With Total Magnetic Resonance Imaging Cerebral Small Vessel Disease Burden

**DOI:** 10.3389/fnagi.2021.640844

**Published:** 2021-05-12

**Authors:** Yutong Hou, Yue Li, Shuna Yang, Wei Qin, Lei Yang, Wenli Hu

**Affiliations:** Department of Neurology, Beijing Chao-Yang Hospital, Capital Medical University, Beijing, China

**Keywords:** cerebral small vessel disease, total cerebral small vessel disease burden, gait, upper extremities, motor performance

## Abstract

**Background and Purpose:** Cerebral small vessel disease (cSVD)—including white matter hyperintensities (WMHs), cerebral microbleeds (CMBs), lacunes, and enlarged perivascular spaces (EPVS)—is related to gait impairment. However, the association between the total magnetic resonance imaging (MRI) cSVD burden and gait and upper extremity function remains insufficiently investigated. This study aimed to assess the correlation between the total MRI cSVD burden score and gait impairment as well as upper extremity impairment.

**Method:** A total of 224 participants underwent MRI scans, and the presence of lacunes, WMHs, CMBs, and EPVS was evaluated and recorded as a total MRI cSVD burden score (range 0–4). Gait was assessed by 4-m walkway, Tinetti, Timed Up and Go (TUG), and Short Physical Performance Battery (SPPB) tests. Upper extremity function was assessed by 10-repeat hand pronation-supination time, 10-repeat finger-tapping time, and 10-repeat hand opening and closing time.

**Result:** The mean age of the 224 participants was 60.6 ± 10.5 years, and 64.3% were men. Independent of age, sex, height, and vascular risk factors, multivariable linear regression analyses showed that a higher total MRI cSVD burden score was related to a shorter stride length, wider step width, higher cadence, and poorer performance on the Tinetti, TUG, and SPPB tests and upper extremity tests (all *P* < 0.05).

**Conclusion:** Total MRI cSVD burden was associated with gait impairment and upper extremity disturbances, suggesting that total MRI cSVD burden might contribute to motor function decline. Longitudinal studies are required to determine whether there is a causal relationship between total MRI cSVD burden and motor function decline.

## Introduction

Cerebral small vessel disease (cSVD) refers to a group of pathological processes, including white matter hyperintensities (WMHs), lacunes, enlarged perivascular spaces (EPVS), cerebral microbleeds (CMBs) and brain atrophy, that are identified by magnetic resonance imaging (MRI) (Debette et al., 2019). Several studies have demonstrated that WMHs are associated with gait abnormalities (Rosano et al., 2008, 2010; Soumaré et al., 2009; de Laat et al., 2010a,b; Srikanth et al., 2010; Pasi et al., 2016). Other cross-sectional analyses have confirmed the relationship among brain atrophy, CMBs lacunes, and gait impairment (de Laat et al., 2011; Stijntjes et al., 2016). However, the relationship between each cSVD marker and gait performance remains unclear.

Compared to gait impairment, research on upper extremity functions is rare. Limited studies have indicated that impaired hand motor functions in the aging population are related to a decline in independent living ability and increased mortality (Scherder et al., 2008). A recent study explored the relationships between each individual cSVD marker and upper extremity function and showed that higher WMH burden and brain atrophy were significantly associated with deficits in upper extremity movement (Su et al., 2017).

However, most of the studies have focused on individual MRI features, and only a few studies have explored the association between total MRI cSVD burden and motor impairment; thus, this association needs more investigation. Staals et al. (2014) proposed a scale to evaluate comprehensive cSVD burden by summing different MRI features, including WMHs, lacunes, CMBs and EPVS. The resulting total MRI cSVD burden score may be a more appropriate method to represent a comprehensive overview of cSVD-related brain damage.

In our study, we aimed to investigate the cross-sectional relationship between the total MRI cSVD burden and gait as well as upper extremity impairment.

## Subjects and Methods

### Study Population

We recruited consecutive participants presenting for physical examination at the Department of Neurology in Beijing Chao-Yang Hospital, Capital Medical University, from February 2019 to August 2019. The exclusion criteria were as follows: (1) dementia; (2) Parkinson's disease; (3) intracranial hemorrhage; (4) intracranial space-occupying lesion; (5) cognitive impairment; (6) recent or current use of acetylcholine esterase inhibitors, neuroleptic agents, and/or L-dopa; (7) severe arthritis or psychogenic gait disturbance; and (8) prominent visual or hearing impairment.

### Ethics Statement

All participants consented to participate in our study and signed an informed consent to the use of data for research. The design of this study was approved by the Ethics Committee of Beijing Chao-Yang Hospital, Capital Medical University, and the study was performed in accordance with the Declaration of Helsinki.

### Clinical Data Collection

Demographic information (age, sex, height, and weight) and vascular risk factors (hypertension, systolic blood pressure, diastolic blood pressure, diabetes mellitus, coronary artery disease, dyslipidemia, and alcohol and/or tobacco use) were recorded and analyzed. Laboratory parameters were acquired the next morning on an empty stomach.

### Motor Performance Assessment

Quantitative gait analysis was performed with a 4-m walkway, and the participants walked twice at self-selected normal gait speed in low-heeled shoes. They started 2 m before the walkway and walked until 2 m beyond it to measure steady-state walking. We recorded velocity (m/s), stride length (m; the distance between the heel points of two consecutive footprints), cadence (number of steps on the 4-m walkway), and stride width (cm; the distance between one midpoint of a footprint and the line of progression of the opposite foot) and recorded the average values. Semiquantitative assessment consisted of the Tinetti test, TUG test, and SPPB test. The upper extremity functions were assessed by 10-repeat hand pronation-supination time, 10-repeat finger-tapping time, and 10-repeat hand opening and closing time.

### MRI

All participants underwent brain MRI on a 3T MRI scanner (Prisma; Siemens AG, Erlangen, Germany). Sequences included diffusion-weighted, T1-weighted, and T2-weighted imaging; fluid-attenuated inversion recovery (FLAIR); and susceptibility-weighted imaging (SWI). The MRI markers of cSVD, including lacunes, WMHs, CMBs, and EPVS, were rated according to the Standards for Reporting Vascular Changes on Neuroimaging (STRIVE) consensus criteria (Gregoire et al., 2009). Deep and periventricular WMHs were graded using the Fazekas scale (Wardlaw et al., 2013). CMBs were defined as round or ovoid lesions of ≤10 mm with low signal intensity on SWI and categorized according to the Microbleed Anatomical Rating Scale (Fazekas et al., 1987). Lacunes were defined as round or ovoid fluid-filled cavities of 3–15 mm on T2-W and FLAIR(Wardlaw et al., 2013). EPVS were identified as punctate or linear hyperintensities on T2-W images in the basal ganglia (BG) or centrum semiovale (CSO). A 4-point visual rating ordinal scale (0, no EPVS; 1, ≤10 EPVS; 2, 11–20 EPVS; 3, 21–40 EPVS; and 4, >40 EPVS) was used to evaluate the severity of EPVS (Doubal et al., 2010).

We used the recently reported scale to represent the total MRI cSVD burden by counting the presence of each of the four features of cSVD (Staals et al., 2014). A point was awarded for each of the following items: ≥1 lacune; Fazekas score ≥2 in deep WMH and/or Fazekas score of 3 in periventricular WMH; ≥1 deep or infratentorial CMB (Poels et al., 2010); moderate to extensive (grade 2–4) EPVS in the BG (Doubal et al., 2010). Hence, the score ranged from 0 to 4 points.

### Statistical Analysis

Continuous variables with a normal distribution were presented as the mean with standard deviation (SD), and variables with a non-normal distribution were presented as the median with interquartile range. We compared the clinical information and imaging characteristics using one-way analysis of variance and Kruskal–Wallis test for continuous data and chi-square test or Fisher exact test for categorical data. The association between motor performance and total MRI cSVD burden was examined using Spearman correlation analysis. Multivariable linear regression analyses were used to adjust for age, sex, height, and vascular risk factors. Statistical significance was established at *P* < 0.05. All analyses were performed using SPSS software (version 24).

## Results

### Characteristics

A total of 275 participants were recruited, but 51 were excluded (21 participants had a history of stroke, 9 participants had incomplete MRI, and the other participants were unable to complete all motor function tests). In the end, a total of 224 participants with an average age of 60.6 years (35–85 years) who underwent brain MRI and motor tests were enrolled in the original cohort, and 144 (64.3%) were male. For total MRI cSVD burden, 52 (32.1%) participants had a score of 0; 72 (32.1%) had a score of 1; 56 (25.0%) had a score of 2; 36 (16.1%) had a score of 3; and 8 (3.6%) had a score of 4. The clinical characteristics of the participants are presented in Table 1, and there was a difference in age. Table 2 shows information on gait and upper extremity function, and significant differences were found. Laboratory tests are presented in Supplementary Table 1, and increased neutrophil counts were found in groups with a high cSVD burden.

**Table 1:** Demographic and clinical characteristics of participants with different severity of total MRI cSVD burden.

**Variables**	**All** **(*n* = 224)**	**cSVD 0** **(*n* = 52)**	**cSVD 1** **(*n* = 72)**	**cSVD 2** **(*n* = 56)**	**cSVD 3** **(*n* = 36)**	**cSVD 4** **(*n* = 8)**	***P***
Age, year	60.6 ± 10.5	56.54 ± 11.1	58.3 ± 11.5	63.5 ± 7.5	64.2 ± 8.1	69.8 ± 8.8	**0.000**
Sex, male, n (%)	144.0 (64.3)	33 (63.5)	43 (59.7)	40 (71.4)	22 (61.1)	6 (75.0)	0.650
Height, m	166.6 ± 8.2	167.4 ± 8.3	166.7 ± 8.1	165.3 ± 8.1	166.6 ± 8.8	169.0 ± 7.4	0.622
Weight, kg	71.6 ± 10.1	71.5 ± 11.1	72.1 ± 10.2	69.7 ± 10.3	74.3 ± 8.6	70.3 ± 7.6	0.323
BMI, kg/m^2^	25.8 ± 3.9	25.4 ± 3.4	25.9 ± 2.9	26.0 ± 5.3	26.0 ± 2.5	22.1 ± 6.7	0.046
Hypertension, n (%)	142.0 (63.4)	25 (48.1)	45 (62.5)	40 (71.4)	28 (77.8)	4 (50.0)	0.031
SBP, mmHg	140.0 (129.3,153.8)	137.0 (123.0,147.8)	137.5 (125.3,151.5)	142.5 (132.5,153.0)	145.0 (137.5,157.5)	147.5 (140.0,159.5)	0.577
DBP, mmHg	81.0 (72.3,90.0)	80.0 (70.0,90.0)	80.5 (71.5,87.5)	81.0 (74.5,88.5)	80.5 (73.0,94.5)	89.0 (82.0,97.0)	0.929
DM, *n* (%)	85.0 (37.9)	16 (30.8)	23 (31.9)	26 (46.4)	17 (47.2)	3 (37.5)	0.261
Hyperlipidemia, n (%)	41.0 (18.3)	9 (17.3)	9 (12.5)	12 (21.4)	10 (27.8)	1 (12.5)	0.359
CAD, *n* (%)	39.0 (17.4)	5 (9.6)	13 (18.1)	11 (19.6)	7 (19.4)	3 (37.5)	0.313
TIA, *n* (%)	26.0 (11.6)	5 (9.6)	7 (9.7)	6 (10.7)	8 (22.2)	0 (0.0)	0.240
Smoke, *n* (%)	107.0 (47.8)	23 (44.2)	33 (45.8)	28 (50.0)	16 (44.4)	7 (87.5)	0.223
Alcohol, *n* (%)	86.0 (38.4)	18 (34.6)	27 (37.5)	21 (37.5)	15 (41.7)	5 (62.5)	0.647

*Data represent number (percentage), mean ± standard deviation, or median (interquartile range)*.

*MRI, magnetic resonance imaging; cSVD, cerebral small vessel disease; BMI, body mass index; SBP, systolic blood pressure; DBP, diastolic blood pressure; DM, diabetes mellitus; CAD, coronary artery disease; TIA, transient ischemic attacks*.

*The bold values mean the association between movement data and total cSVD burden is significant*.

**Table 2:** Movement of participants with different severity of total MRI cSVD burden.

**Variables**	**All** **(*n*= 224)**	**cSVD 0** **(*n* = 52)**	**cSVD 1** **(*n* = 72)**	**cSVD 2** **(*n* = 56)**	**cSVD 3** **(*n* = 36)**	**cSVD 4** **(*n* = 8)**	***P***
Stride length, cm	63.3 (58.0,68.0)	67.0 (63.0,73.3)	63.7 (58.0,67.6)	61.6 (54.7,67.9)	60.0 (56.5,67.2)	61.6 (54.5,65.0)	0.001
Stride width, cm	10.8 (8.0,18.0)	7.5 (6.0,10.0)	11.0 (8.0,13.0)	12.0 (10.0,15.0)	13.5 (10.0,15.0)	15.0 (13.3,17.5)	0.000
Cadence, steps	6.3 (5.9,7.0)	6.0 (5.4,6.3)	6.3 (6.0,7.0)	6.6 (5.9,7.3)	6.7 (5.9,7.7)	6.5 (6.2,7.3)	0.001
Gait velocity, m/sec	1.1 (0.9,1.2)	1.2 (1.1,1.2)	1.1 (1.0,1.2)	1.0 (0.9,1.2)	1.0 (0.9,1.1)	0.9 (0.9,1.1)	0.001
Tinetti test	26.0 (24.3,28.0)	28.0 (28.0,28.0)	27.0 (25.0,28.0)	25.0 (24.0,28.0)	24.0 (24.0,26.0)	24.0 (23.0,25.0)	0.000
TUG test, s	9.4 (8.3,11.0)	8.3 (7.6,8.7)	9.2 (8.3,10.2)	10.3 (8.8,11.4)	10.5 (9.5,12.6)	12.2 (11.7,12.4)	0.000
SPPB test	11.0 (9.0,12.0)	12.0 (12.0,12.0)	11.0 (10.0,12.0)	10.0 (9.0,11.0)	9.0 (8.2,11.0)	9.0 (7.3,10.0)	0.000
Left pronation–supination, s	6.8 (5.2,7.7)	5.1 (4.3,6.5)	6.3 (5.2,7.6)	7.1 (6.2,7.7)	7.6 (7.1,8.5)	7.8 (7.2,8.0)	0.001
Right pronation–supination, s	6.6 (5.2,7.7)	5.1 (4.1,6.4)	6.1 (5.1,7.4)	6.9 (6.1,7.8)	7.9 (7.2,8.7)	7.8 (7.2,8.3)	0.002
Mean pronation–supination, s	6.7 (5.3,7.7)	5.1 (4.2,6.5)	6.3 (5.2,7.5)	7.0 (6.2,7.7)	7.8 (7.2,8.6)	7.7 (7.3,8.4)	0.001
Left finger-tapping, s	4.6 (3.9,6.1)	4.0 (3.3,4.9)	4.3 (3.9,5.7)	4.9 (3.9,6.2)	5.9 (4.5,6.7)	6.3 (5.7,7.4)	0.017
Right Finger-tapping, s	4.7 (3.8,6.0)	4.0 (3.3,4.6)	4.4 (3.9,5.9)	5.0 (3.8,6.1)	5.9 (5.1,7.0)	6.6 (5.5,7.2)	0.007
Mean finger-tapping, s	4.6 (3.8,6.1)	3.9 (3.4,4.8)	4.4 (3.9,5.5)	4.9 (3.9,6.2)	5.8 (4.7,6.6)	6.6 (5.7,6.9)	0.008
Left opening and closing hands time, s	4.7 (4.1,5.7)	4.2 (4.0,4.8)	4.7 (4.0,5.3)	4.7 (4.0,5.9)	5.3 (4.8,6.1)	5.2 (3.9,7.1)	0.057
Right opening and closing hands time, s	4.5 (4.0,5.6)	4.1 (4.0,4.6)	4.6 (4.1,5.6)	4.6 (3.9,5.3)	5.4 (4.6,6.1)	5.0 (3.8,7.1)	0.012
Mean opening and closing hands time, s	4.5 (4.1,5.7)	4.2 (4.1,4.7)	4.7 (4.1,5.4)	4.6 (3.8,6.1)	5.4 (4.6,6.2)	5.1 (3.8,7.1)	0.027

*Data represent number (percentage), mean ± standard deviation, or median (interquartile range)*.

*TUG, Timed-Up-and-Go; SPPB, Short Physical Performance Battery*.

### Association Between Total MRI cSVD Burden and Gait Performance

As shown in Table 3, Spearman correlation analysis revealed that gait performance was negatively correlated with the total MRI cSVD burden. After adjustments for age, sex, and height (model 1, Table 3), total cSVD burden was positively related to stride width (β = 1.645, *P* < 0.001) and cadence (β = 0.191, *P* = 0.011) and negatively related to stride length (β = −1.458, *P* = 0.002), but there was no association with gait velocity (β = −0.024, *P* = 0.390). After additional adjustment for vascular risk factors (model 2, Table 3), the association was similar. In the semiquantitative assessment, we found that participants with higher total MRI cSVD burden scores had lower Tinetti and SPPB test scores and needed more time to complete the TUG test. In multivariate-adjusted models, the relationships were significant, all *P* < 0.001 (Table 3).

**Table 3:** Association of gait and upper extremities with total MRI cSVD burden.

**Variables**	**Spearman correlation**		**Model 1**		**Model 2**	
	***r***	***P***	**β**	***P***	**β**	***P***
Stride length	−0.265	0.000	−1.458	**0.002**	−1.446	**0.002**
Stride width	0.489	0.000	1.645	**0.000**	1.655	**0.000**
Cadence	0.267	0.000	0.191	**0.011**	0.191	**0.011**
Gait velocity	−0.349	0.000	−0.024	0.390	−0.029	0.336
Tinetti test	−0.558	0.000	−0.913	**0.000**	−0.917	**0.000**
TUG test	0.505	0.000	0.816	**0.000**	0.822	**0.000**
SPPB test	−0.561	0.000	−0.642	**0.000**	−0.651	**0.000**
Left pronation–supination, sec	0.491	0.000	0.597	**0.000**	0.585	**0.000**
Right pronation–supination, sec	0.508	0.000	0.624	**0.000**	0.628	**0.000**
Mean pronation–supination, sec	0.511	0.000	0.611	**0.000**	0.615	**0.000**
Left finger-tapping, sec	0.352	0.000	0.480	**0.000**	0.490	**0.000**
Right Finger-tapping, sec	0.381	0.000	0.495	**0.000**	0.502	**0.000**
Mean finger-tapping, sec	0.379	0.000	0.487	**0.000**	0.496	**0.000**
Left opening and closing hands time, sec	0.255	0.000	0.289	**0.001**	0.287	**0.001**
Right opening and closing hands time, sec	0.259	0.000	0.288	**0.004**	0.289	**0.004**
Mean opening and closing hands time, sec	0.264	0.000	0.289	**0.001**	0.288	**0.001**

*Model 1 is adjusted for gender, age, and height*.

*Model 2 is adjusted for gender, age, height, hypertension, BMI, neutrophil, and triglyceride*.

*The bold values mean the association between movement data and total cSVD burden is significant*.

### Associations Between Total MRI cSVD Burden and Upper Extremity Function

In the baseline analysis, upper extremity performance was assessed by 10-repeat pronation–supination, 10-repeat finger tapping, and 10-repeat opening and closing hands time. In multivariable linear regression, there was a strong correlation between the time of each movement and the total MRI cSVD burden score. There was no significant difference in model 1 and model 2. In addition, we found a similar coefficient for the total MRI cSVD burden between the left and right hands (Table 3).

## Discussion

In this study, we found that total MRI cSVD burden was associated with gait, including stride length, cadence, stride width, and Tinetti, TUG and SPPB scores, but was not associated with gait velocity. In addition, we found novel evidence that the total MRI cSVD burden was related to the function of the upper extremities.

In our study, the difference in age was significantly associated with MRI cSVD burden because age has been shown to be a risk factor for cSVD in many studies. In the analysis of the association between total MRI cSVD burden and movement impairment, age was adjusted for. Our study provided the novel insight that the high cSVD burden group had an increased neutrophil count. A previous study on the pathogenesis of Alzheimer's disease found that neutrophil adhesion in brain capillaries reduced cortical blood flow (CBF) and impaired memory function in mouse models (Cruz Hernández et al., 2019). The increase in neutrophil count might be due to the blockade of neutrophils by narrowed capillaries and inflammation, which were mechanisms of cSVD. In addition, BBB impairment and hypoperfusion were observed in WMHs and normal-appearing white matter (Wong et al., 2019). Another study from Shandong also declared that with the increase in the total cSVD burden score, the global and regional CBF decreased (Yu et al., 2020). This result suggested that the increase in neutrophils induced a reduction in CBF, which is an indicator for the diagnosis of cSVD and may be involved in mechanism of cSVD.

In many previous studies, WMH was believed to be the most important predictor of gait impairment. The LADIS study (Baezner et al., 2008; Kreisel et al., 2013) showed that WMH was associated with the deterioration of gait function, as measured by the SPPB and walking speed. The Three-City (3C) study showed that higher volumes of WMH were associated with slower walking speed and lower Tinetti score independent of lacunar infarcts and cognitive status (Soumaré et al., 2009). The Radboud University Nijmegen Diffusion tensor and MRI Cohort (RUN DMC) study (de Laat et al., 2010b) found that WMH was associated with a shorter stride length in the TUG test but not with velocity, which was consistent with our conclusions. They suggested that stride length is a more sensitive indicator than velocity for gait assessment in cSVD patients and is associated with gait velocity. In many other studies, brain atrophy, especially in the entorhinal cortex, caudate nucleus and basal ganglia, lacunes, and CMBs correlated with worse performance of the lower limbs, but the conclusions were inconsistent (Rosano et al., 2010; de Laat et al., 2011; Stijntjes et al., 2016; Hilal et al., 2017; Sakurai et al., 2018).

To the best of our knowledge, few studies have explored the relationship between cSVD and upper extremities. Upper extremities play an important role in activities of daily living (ADL). With aging, the function of the upper extremities decreases and affects the performance of manual tasks (Simon-Martinez et al., 2018). In a study including a sample of 30 individuals from the Austrian Stroke Prevention Study, upper extremities were evaluated by Purdue's Pegboard Test, and a positive result with cSVD was not found. However, a population-based prospective cohort (Su et al., 2017, 2018) found that WMHs contributed to motor deficits in pronation–supination, and brain atrophy contributed to motor deficits in both pronation–supination and finger tapping. In addition, we also measured opening and closing hands time, which had not been previously measured in this context, and obtained a positive result. This provided a new method for the measurement of upper limb function in cSVD.

Although these studies have shown that individual MRI markers of cSVD are independently linked to concurrent gait impairment, these cSVD markers rarely occur in isolation on MRI. There is increasing evidence that a total cSVD MRI burden score summarizing individual cSVD markers on a compound scale might better reflect the overall effect of cSVD on the brain and be more representative of cSVD (Gregoire et al., 2009; Huijts et al., 2013; Pinter et al., 2017). Our research showed that as the total MRI cSVD burden score increased, the motor index gradually deteriorated, and the correlation was significant.

Studies focused on the association between individual cSVD MRI markers and movement impairment are common, but studies on total MRI cSVD burden are rare. One study (Hatate et al., 2016) showed that gait function, as assessed by the Unified Parkinson's Disease Rating Scale, was associated with total cSVD burden. This was the first study focused on gait function and overall cSVD burden, but EPVS was not included in the total MRI cSVD burden score. Another study suggested that the total cSVD burden and lacunes in the basal ganglia might independently contribute to gait dysfunction in Parkinson's disease, although gait was evaluated by a semiquantitative assessment that was probably unable to detect a change in gait (Chen et al., 2020). Recently, a study in community-dwelling older subjects reported that total MRI cSVD burden was associated with lower stroke impact scale (SIS) mobility domain scores in non-lacunar stroke patients but not lower TUG test scores, and the gait parameters were not measured (Loos et al., 2017). To the best of our knowledge, the relationship between total MRI cSVD burden and upper extremity impairment has never been studied. In our study, we found that a higher total MRI cSVD burden score was associated with impaired upper extremity performance. In addition, we applied quantitative and semiquantitative methods to detect abnormal gait, and the participants with higher total MRI cSVD burden scores were more likely to have worse gait function.

The mechanisms linking cSVD and motor function are still not well-understood. Some studies (de Laat et al., 2010b; Linortner et al., 2012) have suggested that WMH might disrupt long loop reflexes consisting of deep white matter sensory and motor tracts, especially the tissue located in frontal regions, which controlled motor function. The findings of one study showed that gray and white matter atrophy resulting in motor disturbances could be a pathological consequence of subcortical lesions, which agreed with the view above (Su et al., 2018). Further research using diffusion tensor imaging (DTI) found that the loss of microstructural integrity of fibers in the cingulum, the genu of the corpus callosum, and the inferior longitudinal fasciculus led to a lower gait velocity and a reduced stride length (Pasi et al., 2016; Rosario et al., 2016). Furthermore, the volumetric loss of deep subcortical nuclei and anterior cingulate cortex involved in the locomotion network was reported (de Laat et al., 2012). WMH was confirmed to be associated with brain atrophy and to lead to motor function impairment. In addition to the cortical and subcortical regions, Karlijn demonstrated that people with WMHs in the brain stem were associated with a lower cadence, suggesting that cadence was regulated by the brain stem (and cerebellum), such as the mesencephalic locomotor region (de Laat et al., 2010b). Recent studies also found that brain networks that controlled rhythm and pace were linked to networks involved in the performance of verbal fluency tasks during semantic dual-tasking, and results from older people with dementia aligned with these findings (Taylor et al., 2019). Lacunes and CMBs were considered to disrupt the connections of basal ganglia-thalamofrontal cortical circuits and damage neural networks (Hatate et al., 2016). A recent study indicated that CMBs in the temporal lobe, which processes visual and vestibular signals, affected gait performance (de Laat et al., 2011).

Takakusaki (2017) suggested that movement of the upper extremities was a complex ability dependent on the integration of the visual, somatosensory, and action systems of the motor cortex and its subcortical connectivity with the brainstem and cerebellum. Many studies have reported age-related declines in different upper extremity sensorimotor parameters, such as motor coordination, manual dexterity, strength, and sensibility (Joseph et al., 2017; Simon-Martinez et al., 2018). The mechanism is still unclear. A recent study found that central motor conduction time (CMCT), an indicator of pyramidal tract dysfunction in motor-evoked potentials (MEPs), was associated with the severity of EPVS in the white matter, and the study of Xinhua Hospital found a similar result: the tremor score was associated with the EPVS score (Wan et al., 2019). We speculated that the pathophysiological changes caused by EPVS might contribute to dysfunction of the upper extremities.

Strengths of our study are that (1) we used total MRI cSVD burden, a more representative and comprehensive method to reflect the severity of cSVD, to analyze the association between cSVD and gait disorder; (2) we found that the inclusion of damage in the upper extremities in the quantitative evaluation of total MRI cSVD burden had an important impact on diagnosis; and (3) the novel finding of increased neutrophil number in patients with high total MRI cSVD burden may provide a new direction in therapy.

Some limitations should be mentioned. First, because the participants were selected from a single center, the generalizability of the results to the general population might be limited. Second, although few studies have investigated whether the total MRI cSVD burden score is a possible marker of gait and upper extremity impairment, our sample size is small, and large-scale research is needed. Third, brain atrophy may influence motor function, but it was not included in the calculation of the total MRI cSVD burden score. Fourth, our study did not reveal whether the total MRI cSVD burden can predict the progression of motor function impairment, and longitudinal studies are needed.

## Conclusion

The present study indicated that total MRI cSVD burden was associated not only with quantitative data like stride length, cadence, and stride width but also semiquantitative data including TUG, Tinetti, and SPPB test results. A new view of the relationship between total MRI cSVD burden and upper extremity impairment was found. An interesting finding of increased neutrophil number in individuals with high total MRI cSVD burden was shown. Further research is needed to confirm these results, and these factors should be considered in new strategies for intervention.

## Data Availability Statement

The raw data supporting the conclusions of this article will be made available by the authors, without undue reservation.

## Ethics Statement

All participants consented to participate in our study and signed an informed consent to the use of data for research. The design of this study was approved by the Ethics Committee of Beijing Chao-Yang Hospital, Capital Medical University, and the study was performed in accordance with the Declaration of Helsinki.

## Author Contributions

YH planned the study, collected data, and wrote the manuscript. YL and WQ collected the data and revised the manuscript. SY and YH analyzed the data and revised the manuscript. SY, YH, and WQ interpreted the data and revised the manuscript. WH designed the study and revised the manuscript. All authors read and approved the final manuscript.

## Conflict of Interest

The authors declare that the research was conducted in the absence of any commercial or financial relationships that could be construed as a potential conflict of interest.
