# Supplementary material for: Gait Impairment and Upper Extremity Disturbance Are Associated With Total Magnetic Resonance Imaging Cerebral Small Vessel Disease Burden
Source: Front Aging Neurosci. 2021 May 12;13:640844. doi: 10.3389/fnagi.2021.640844 (PMC8149961; doi:10.3389/fnagi.2021.640844)
Supplement: Supplementary file 1 [file Table_1.doc]

Supplementary Material Table S1 Laboratory tests in participants with different severity of total MRI cSVD burden

| Variables | All  (n= 224) | cSVD 0  (n = 52) | cSVD 1  (n =72 ) | cSVD 2  (n =56) | cSVD 3  (n =36 ) | cSVD 4  (n =8 ) | *P* |
| --- | --- | --- | --- | --- | --- | --- | --- |
| WBC, *10^9^/L | 6.3 (5.5,7.6) | 6.3 (5.3,7.4) | 5.9 (5.4,7.1) | 6.6 (6.1,7.8) | 6.7 (5.2,7.6) | 6.6 (5.7,8.8) | 0.649 |
| Neutrophil,% | 60.1±10.6 | 57.2±9.7 | 58.7±8.2 | 62.1±8.8 | 61.2±9.4 | 72.6±29.2 | **0.001** |
| Hemoglobin, g/L | 142.0 (132.0,150.0) | 142.0 (131.0,149.5) | 142.0 (131.5,150.0) | 141.0 (132.0,149.0) | 140.0 (134.0,149.0) | 142.5 (130.5,187.5) | 0.929 |
| Platelet count, *10^9^/L | 208.5 (175.0,250.0) | 217.5 (174.3,263.3) | 206.0 (170.0,250.0) | 211.0 (177.0,242.0) | 219.0 (182.0,262.0) | 199.5 (172.8,256.5) | 0.530 |
| Cholesterol,mmol/L | 4.4 (3.8,5.2) | 4.4 (3.8,5.1) | 4.7 (3.8,5.4) | 4.3 (3.8,5.2) | 4.4 (3.5,5.0) | 4.5 (3.3,5.4) | 0.452 |
| LDL, mmol/L | 2.5±0.9 | 2.5±0.8 | 2,5±0.9 | 2.5±0.8 | 2.5±0.9 | 2.7±1.0 | 0.985 |
| HDL, mmol/L | 1.0 (0.9,1.2) | 1.0 (0.8,1.2) | 1.0 (0.9,1.2) | 1.0 (0.9,1.3) | 1.0 (0.9,1.2) | 1.0 (0.9,1.2) | 0.788 |
| Triglyceride, mmol/L | 1.5 (1.2,2.2) | 1.3 (1.1,1.9) | 1.7 (1.3,2.4) | 1.5 (1.0,2.0) | 1.5 (1.2,1.8) | 1.7 (1.3,2.4) | 0.033 |
| AST, U/L | 20.0 (18.0,25.0) | 20.5 (18.0,25.3) | 21.0 (17.0,25.0) | 20.0 (18.0,25.0) | 19.0 (17.0,22.0) | 19.0 (16.5,2280) | 0.933 |
| ALT,U/L | 19.5 (15.0,26.0) | 19.0 (15.8,29.0) | 19.0 (15.0,26.0) | 21.0 (15.0,27.0) | 17.0 (13.0,21.0) | 19.5 (13.0,23.0) | 0.775 |
| ALP, U/L | 71.0 (60.0,84.8) | 68.0 (61.5,81.0) | 72.0 (62.5,87.5) | 70.0 (60.0,83.0) | 73.0 (58.0,70.0) | 80.0 (61.5,97.3) | 0.428 |
| Fasting glucose, mmol/L | 5.6 (4.8,6.9) | 5.7 (4.8,7.9) | 5.3 (4.7,6.2) | 5.8 (4.8,7.1) | 5.6 (4.9,6.7) | 7.1 (6.2,8.3) | 0.308 |
| HbA1C, % | 6.0 (5.6,7.0) | 6.0 (5.7,7.1) | 5.8 (5.6,6.3) | 6.1 (5.5,7.3) | 6.0 (5.7,7.1) | 7.3 (6.6,8.4) | 0.242 |
| Urea, mmol/L | 4.9 (4.1,5.8) | 4.8 (4.0,5.8) | 5.0 (4.1,5.7) | 4.8 (4.1,5.9) | 5.0 (4.6,5.6) | 4.3 (3.8,7.9) | 0.907 |
| Creatinine,umol/L | 63.5 (54.1,73.1) | 61.8 (49.8,72.1) | 61.0 (51.6,71.4) | 65.9 (58.8,77.6) | 63.5 (55.4,69.5) | 78.3 (66.7,105.3) | 0.869 |
| Uric acid，umol/L | 325.0±26.4 | 329.1±100.1 | 309.2±96.6 | 355.4±92.8 | 303.4±78.1 | 325.8±130.3 | 0.050 |
| Natrium, mmol/L | 140.5 (139.2,142.1) | 140.5 (139.3,141.9) | 141.0 (139.5,142.3) | 140.1 (139.2,141.7) | 140.5 (139.5,142.2) | 138.9 (138.6,141.8) | 0.450 |
| [Kalium](../../../../C:/Users/houyutong/Desktop/manuscript(2)%E4%BF%AE%E6%94%B9.doc#/javascript:;), mmol/L | 4.0 (3.7,4.2) | 4.0 (3.7,4.2) | 3.9 (3.7,4.1) | 4.1 (3.7,4.3) | 4.0 (3.8,4.2) | 4.0 (3.9,4.1) | 0.423 |
| Homocysteine, umol/L | 13.0 (11.0,16.0) | 14.0 (10.0,17.0) | 13.0 (11.0,16.0) | 13.0 (11.0,16.0) | 13.0 (11.0,17.0) | 13.5 (10.6,19.5) | 0.849 |
| PT, sec | 11.2 (10.7,11.7) | 11.8 (11.2,12.4) | 11.7 (11.2,12.2) | 11.6 (11.2,12.0) | 11.6 (11.1,12.1) | 11.5 (11.0,12.3) | 0.835 |
| Fibrinogen, mg/dl | 243.0 (209.9,285.2) | 243.0 (207.5,277.2) | 240.1 (208.2,281.2) | 242.9 (202.8,296.5) | 251.9 (215.6,290.3) | 269.7 (193.0,294.2) | 0.921 |
| INR | 0.9 (0.9,1.0) | 0.9 (0.9,1.0) | 0.9 (0.9,1.0) | 0.9 (0.9,1.0) | 0.9 (0.9,0.9) | 0.9 (0.9,1.0) | 0.422 |
| D-dimer, mg/L | 0.2 (0.1,0.4) | 0.2 (0.2,0.3) | 0.2 (0.1,0.4) | 0.3 (0.1,0.6) | 0.2 (0.1,0.4) | 0.4 (0.3,0.8) | 0.798 |

Data represent number (percentage), mean ± standard deviation, or median (interquartile range).

WBC, [white](../../../../C:/Users/houyutong/Desktop/manuscript(2)%E4%BF%AE%E6%94%B9.doc#/javascript:;) [blood](../../../../C:/Users/houyutong/Desktop/manuscript(2)%E4%BF%AE%E6%94%B9.doc#/javascript:;) [cell](../../../../C:/Users/houyutong/Desktop/manuscript(2)%E4%BF%AE%E6%94%B9.doc#/javascript:;); PT, prothrombin time; INR, international normalized ratio; AST, aspartate aminotransferase; ALT, alanine transaminase; ALP, alkaline phosphatase; LDL, low-density lipoprotein cholesterol; HDL, high-density lipoprotein cholesterol
